# AI-based reconstruction from low-resolution acquisitions for diffusion MRI: evaluation of FOD similarity

**DOI:** 10.1007/s12194-026-01103-4

**Published:** 2026-08-07

**Authors:** Akihiro Kasahara, Yuichi Suzuki, Kazuki Endo, Ryosuke Mori, Ryutaro Yano, Hiroshi Kusahara, Toshihiro Hayashi, Osamu Abe

**Affiliations:** 1 Radiology Center, The University of Tokyo Hospital, Tokyo, Japan; 2 Canon Medical Systems Corporation, Tochigi, Japan; 3 Department of Radiology, The University of Tokyo Hospital, Tokyo, Japan

**Keywords:** Magnetic resonance imaging, Diffusion-weighted Imaging, High angular resolution diffusion imaging, Artificial intelligence, Super-resolution

## Abstract

MRI provides essential insights into tissue microstructure, and high angular resolution diffusion imaging (HARDI) enables detailed assessment of complex white matter architecture through fiber orientation distribution (FOD) analysis. However, HARDI requires high b-values and multiple diffusion directions, leading to reduced signal-to-noise ratio (SNR) and long scan times. Conventional zero-fill interpolation processing (ZIP) is widely used for super-resolution but is limited by edge blurring and artifacts. This study evaluated an AI-based reconstruction method, Precise IQ Engine (PIQE). Specifically, low-resolution diffusion data were reconstructed to standard resolution and compared with standard-resolution acquisitions. Ten healthy volunteers underwent HARDI at 3T, and FOD were estimated using constrained spherical deconvolution. Quantitative comparisons with reference data demonstrated that PIQE exhibited higher distributional similarity (lower Jensen–Shannon divergence) and directional agreement (higher angular correlation coefficient) compared with ZIP+Advanced Intelligent Clear-IQ Engine (AiCE), with statistically significant similarity observed. These findings indicate potential usefulness in advanced diffusion MRI applications.

## Introduction

Magnetic resonance imaging (MRI) is a vital noninvasive technique for visualizing the structural and functional properties of biological tissues [1]. Diffusion-weighted imaging (DWI) provides a reliable method for characterizing water diffusion in vivo [2]. To further investigate tissue microstructure, several model-based approaches have been developed. However, conventional diffusion tensor imaging (DTI) often fails to resolve complex architectures, particularly in brain regions with crossing fibers [3].

To address this, high angular resolution diffusion imaging (HARDI) was developed, enabling detailed evaluation of multiple fiber populations. From HARDI data, the fiber orientation distribution (FOD) can be reconstructed to provide voxel-wise representations of complex fiber orientations [4]. Constrained spherical deconvolution (CSD) is a widely used technique for FOD estimation [5], as it deconvolves the diffusion signal with a response function of a single fiber population. This overcomes the limitations of tensor models and is crucial for advanced applications such as tractography and fixel-based analysis, providing insights into white matter integrity and connectivity.

Despite its advantages, HARDI requires high b-values and numerous motion probing gradients (MPG), leading to decreased signal-to-noise ratio (SNR) and prolonged scan times [6]. To mitigate these challenges, recent research has focused on artificial intelligence (AI)-based reconstruction [7]. While conventional methods like zero-fill interpolation processing (ZIP) are computationally efficient, they lack actual high-frequency information, resulting in blurring and ringing artifacts.

In contrast, a recently proposed method, Precise IQ Engine (PIQE), leverages a two-stage neural network to simultaneously achieve noise reduction and super-resolution [8]. The first network enhances the SNR, while the second specifically addresses the limitations of ZIP-processed images to suppress blurring. In this study, to ensure a fair comparison, PIQE is compared against ZIP combined with AiCE, another AI-based denoising technique [7]. Both methods likely rely on deep learning-based reconstruction (dDLR) principles [9]. The purpose of this study is to evaluate the similarity of fiber orientation estimation in HARDI by comparing the newly developed PIQE with conventional ZIP techniques.

## Materials and methods

### Subjects

The present study was approved by the Ethics Committee of The University of Tokyo Hospital (Approval Number: 10727-(6)). Ten healthy adult volunteers (10 males; mean age: 33.00 years, SD: 2.75 years) with no history of neurological or psychiatric disorders provided written informed consent.

### Imaging parameters

MR examinations were performed on a clinical 3-T system (Vantage Centurian, Canon Medical Systems, Otawara, Japan;100 mT/m max. gradient strength, 200T·m-1·s-1max. slew rate) using a 32-channel head coil. We collected:

- Reference data: Standard resolution DWI.
- Original data: Half-matrix resolution of the reference data to which reconstruction techniques were applied; these are referred to as ZIP+AiCE data and PIQE data.
- 3D T1 weighted image (3D-T1WI): For anatomical reference.

*The reference data*: Single-shot spin-echo echo planar imaging; repetition time (TR)/echo time (TE) = 5650/74 msec; flip angle = 90°; slice thickness/gap = 2.5/0 mm; number of slices = 60; field of view (FOV) = 256 × 256 mm^2^; matrix = 128 × 128; voxel size = 2 × 2 × 2.5 mm^3^; b values = 0,3000 s/mm^2^; motion probing gradient (MPG) = 64 directions (electrostatic repulsion model); in-plane acceleration factor (SPEEDER factor) = 2; slice acceleration factor (multi-band SPEEDER factor) = 2; acquisition time = 407 s. Under identical scan time conditions, the 64 × 64 matrix acquisition yields inherently higher per-voxel SNR than the 128 × 128 reference acquisition, owing to the reduced number of k-space lines sampled per unit voxel volume. In addition, b_0_ images with the reversed phase encoding direction were also acquired for distortion correction processing.

*The original data*: Matrix = 64 × 64; voxel size = 4 × 4 × 2.5 mm^3^, and acquisition time = 407 s. Other parameters were set to the same as those for the reference data. The acquired data was processed using ZIP with AiCE or PIQE, reconstructed the same as the reference data (matrix = 128 × 128; voxel size = 2 × 2 × 2.5 mm). In the present study, to ensure a fair comparison and minimize bias between methods, ZIP was used in combination with AiCE. The denoising neural network performed in PIQE’s first stage is based on an AI algorithm functionally similar to AiCE.

*The 3D-T1WI*: Inversion recovery-prepared 3D gradient echo; TR/TE = 7/2 msec; flip angle = 10°; slice thickness = 1 mm, number of slices = 176; FOV = 256 × 256 mm^2^; matrix = 256 × 256; voxel size = 1 × 1 × 1 mm^3^; inversion time = 900 msec; SPEEDER factor = 2; scan time = 165 s.

### Preprocessing

**Fig. 1:**
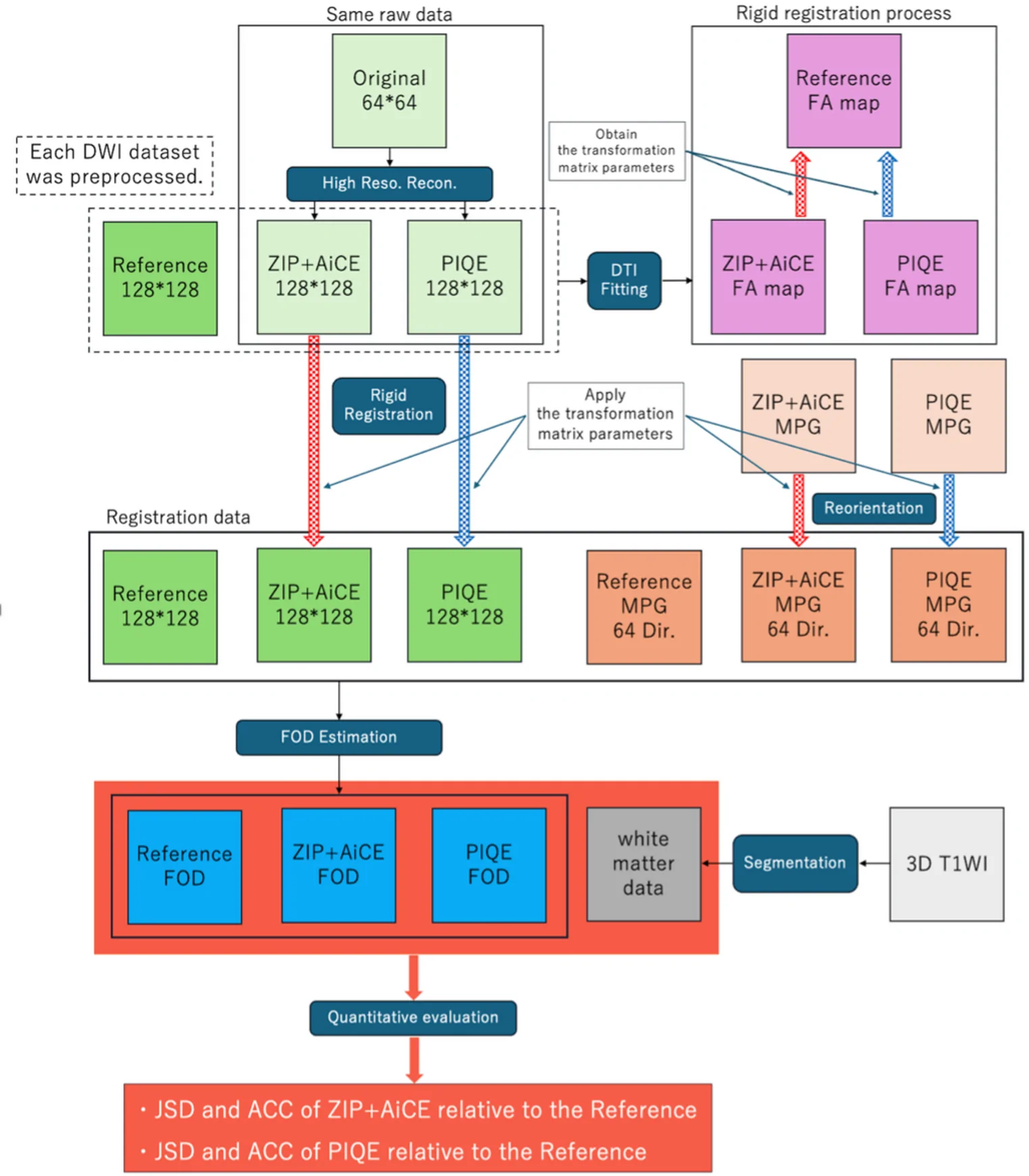
An overview of the entire workflow, from image reconstruction to quantitative evaluation

An overview of the entire workflow, from image reconstruction to quantitative evaluation, is shown in Fig. 1.

We performed noise removal and ringing artifact removal using MRtrix3 (version 3.0_RC3) [10], distortion and motion correction using FSL (version 6.0.6.2) with the top-up and eddy tools [11], and then B_1_ field correction using MRtrix3. Subsequently, fractional anisotropy (FA) maps were computed from the reference data using the diffusion tensor model and served as the reference standard. FA maps were also calculated from the ZIP and PIQE data and spatially aligned to the reference FA map with a rigid-body transformation using FSL with the FLIRT tool. Since diffusion at high b-values (e.g., b = 3000 s/mm²) enters a non-monoexponential regime, the FA maps derived from these data were not considered for quantitative interpretation. Instead, these FA maps were used solely as registration contrasts to improve spatial alignment between datasets. The same registration parameters obtained from the FA alignment were then applied to the ZIP and PIQE data themselves, aligning them to the reference data. Furthermore, white matter mask was generated by segmentation of 3D T1WI using FSL with FAST tool and co-registered with the DWI data to enable white matter-specific analysis.

To ensure the validity of FOD metrics, we quantitatively evaluated the rotation magnitude during the registration between the Reference and the reconstructed datasets. The total rotation angles were found to be very small, with a mean of 0.20° and a maximum of 1.28° across all subjects. Furthermore, since the b-matrix (gradient directions) was mathematically reoriented for each case to match the rotation, the impact on the orientation-dependent parameters was considered negligible. Given that these rotation angles are substantially smaller than the angular interval of our diffusion encoding and have been rigorously compensated for, we believe that these minor residual variations do not confound the interpretation of JSD and ACC values. Therefore, the validity of our FOD comparison remains robust.

### FOD estimation

This evaluation quantitatively assessed how closely each method(ZIP+AiCE data and PIQE data) could reconstruct images that approximate the reference data. Particular attention was given to the similarity of white matter structure estimation relative to the reference data in diffusion MRI, focusing on how well each method preserved fine-scale anatomical features [12]. Specifically, FOD were computed from the diffusion signal using CSD, with the distributions represented by spherical harmonics (SH) [4]. For each reconstruction method, the response functions were estimated individually using the iterative Tournier algorithm. A common response function was not used, as it could introduce an artificial bias by imposing a suboptimal template on one of the methods. By calibrating the response function to the signal characteristics of each dataset, each reconstruction technique was evaluated under its respective optimal conditions. The reconstructed FOD were then quantitatively compared with those obtained from the reference data to assess the similarity. FOD is defined as [4, 5]:

$$\:FOD\left(\theta\:\:,\:\varphi\:\right)={\sum\:}_{l=\mathrm{0,2},4,...\:\:}^{L}{\sum\:}_{m=-l}^{l}\:{c}_{lm}{Y}_{lm}(\theta\:,\:\varphi\:)$$

FOD is defined on the spherical coordinates ($\theta ,\,\phi$) and expressed as a linear combination of spherical harmonic functions $Y_{{lm}}$. Here, * l * denotes the degree of the harmonics, *m* indicates the order, and $C_{{lm}}$ represents the coefficient corresponding to each basis function. The maximum degree *L*, also referred to as the band limit, determines the angular resolution of the representation. In this study, the maximum degree L was set to 8.

### Image evaluations

The similarity of ZIP+AiCE data and PIQE data to the reference was evaluated using:

*Jensen-Shannon Divergence (JSD)* [13]: A symmetric measure of FOD distribution similarity. JSD is derived from the Kullback–Leibler (KL) divergence [14], which quantifies the difference between two probability distributions　P and Q:

$$\:KL(P\:\Vert\:\:Q)\:=\:\sum\:_{i}P\left(i\right)\:\:\mathrm{log}\frac{P\left(i\right)}{Q\left(i\right)}$$

where *Pi* and *Qi* represent the i-th element of the 45-dimensional SH coefficient vector derived from the CSD estimation (*N* = 45). While KL divergence is a standard measure of divergence, it is inherently asymmetric, making it unsuitable for balanced mutual similarity assessment. To address this limitation, the JSD was developed as a symmetrized version of KL divergence. JSD ranges from 0 to 1, where 0 indicates that the two distributions are identical:

$$\:JSD(P\:\Vert\:\:Q)\:=\:\frac{1}{2}\:KL(P\:\Vert\:\:M)\:+\:\frac{1}{2}\:KL(Q\:\Vert\:\:M),\:\mathrm{w}\mathrm{h}\mathrm{e}\mathrm{r}\mathrm{e}\:M=\:\frac{1}{2}\:(P+Q)$$

Here, *P* and *Q* represent the normalized FOD distributions from the reference and reconstructed data, respectively. For the quantitative evaluation using JSD, the comparison was performed using the SH representation of the reconstructed FODs. To satisfy the mathematical non-negativity and non-zero requirements of the logarithm in the KL divergence calculation (i.e., avoiding log(negative) or division-by-zero errors in the processing algorithm), a data-filtering protocol was implemented. First, any voxel containing an SH coefficient of exactly zero in any of the 45 elements was excluded from the analysis. Second, for the remaining voxels, the absolute values of the raw SH coefficients were applied temporarily only during the logarithmic summation to ensure computational stability without altering the original dataset.

*Angular Correlation Coefficient (ACC)* [15]: An index measuring the directional agreement between estimated FOD axes, ranging from 0 to 1 (1 indicates perfect agreement):

$$\:ACC\:=\:\frac{{\sum\:}_{i=1}^{N}{P}_{i}\cdot\:{Q}_{i}}{\sqrt{{\sum\:}_{i=1}^{N}{P}_{i}^{2}}\:\:\cdot\:\:\sqrt{{\sum\:}_{i=1}^{N}{Q}_{i}^{2}}}$$

Here, *Pi* and *Qi* represent the i-th element of the 45-dimensional SH coefficient vector derived from the reference and reconstructed target data, respectively, where *N* = 45 corresponds to the total number of SH coefficients for a maximum harmonic degree of L = 8. For the ACC calculation, the raw SH coefficients were utilized as-is, seamlessly preserving their original mathematical meanings, including negative values.

By applying a white matter mask, the JSD and ACC within the white matter were compared for the ZIP+AiCE data and PIQE data.

### Statistics

The Wilcoxon signed-rank test (*p* < 0.05) was applied to the JSD, ACC values [16]. In addition to p-values, effect sizes were calculated using the rank-biserial correlation. Statistical analyses were performed using R (version R 4.5.0) [17].

## Results

**Fig. 2:**
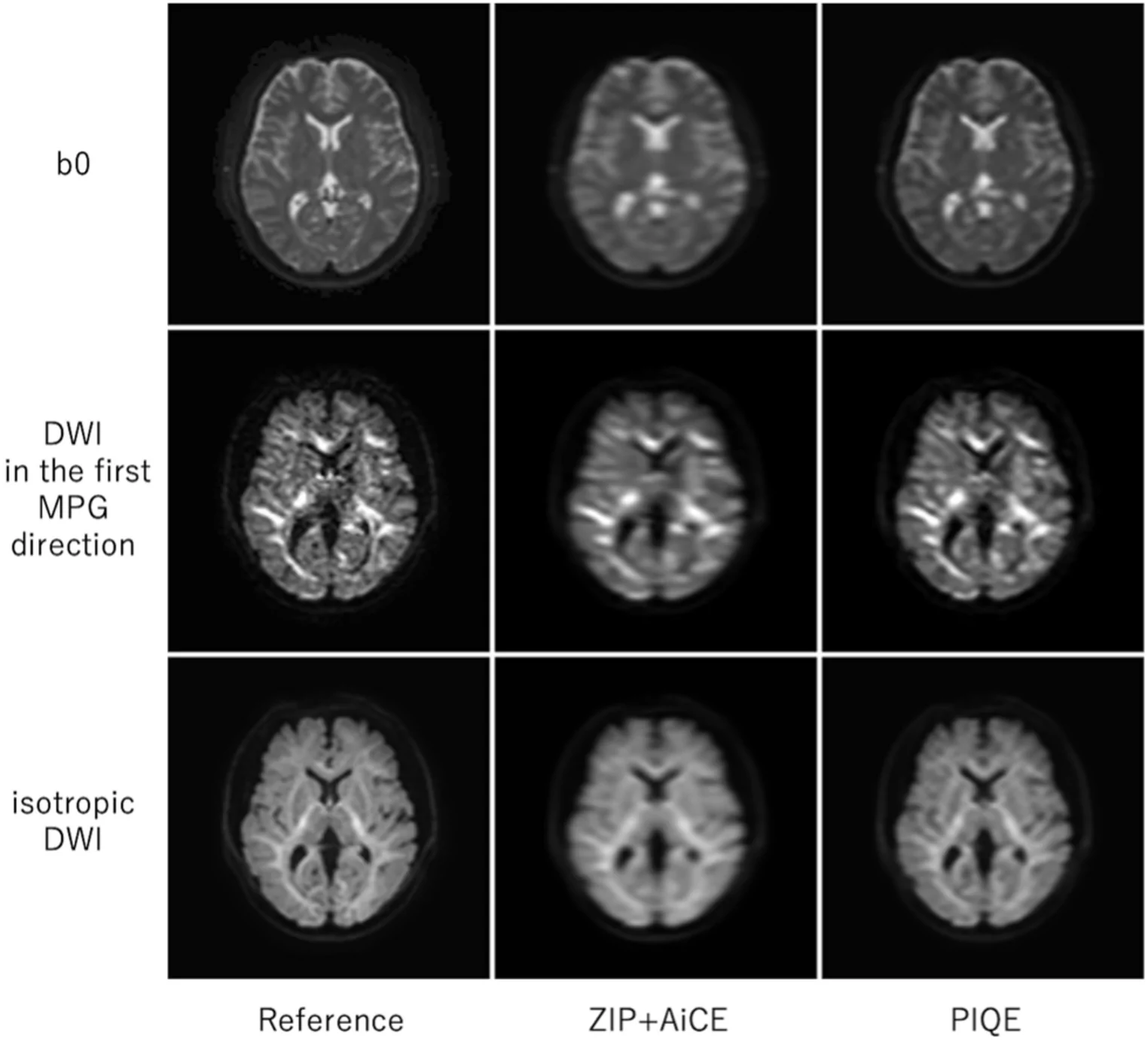
The b0 images, the DWIs acquired in the first of the 64 MPG directions, and the isotropic DWIs reconstructed from the reference, ZIP+AiCE, and PIQE data for a representative subject

**Fig. 3:**
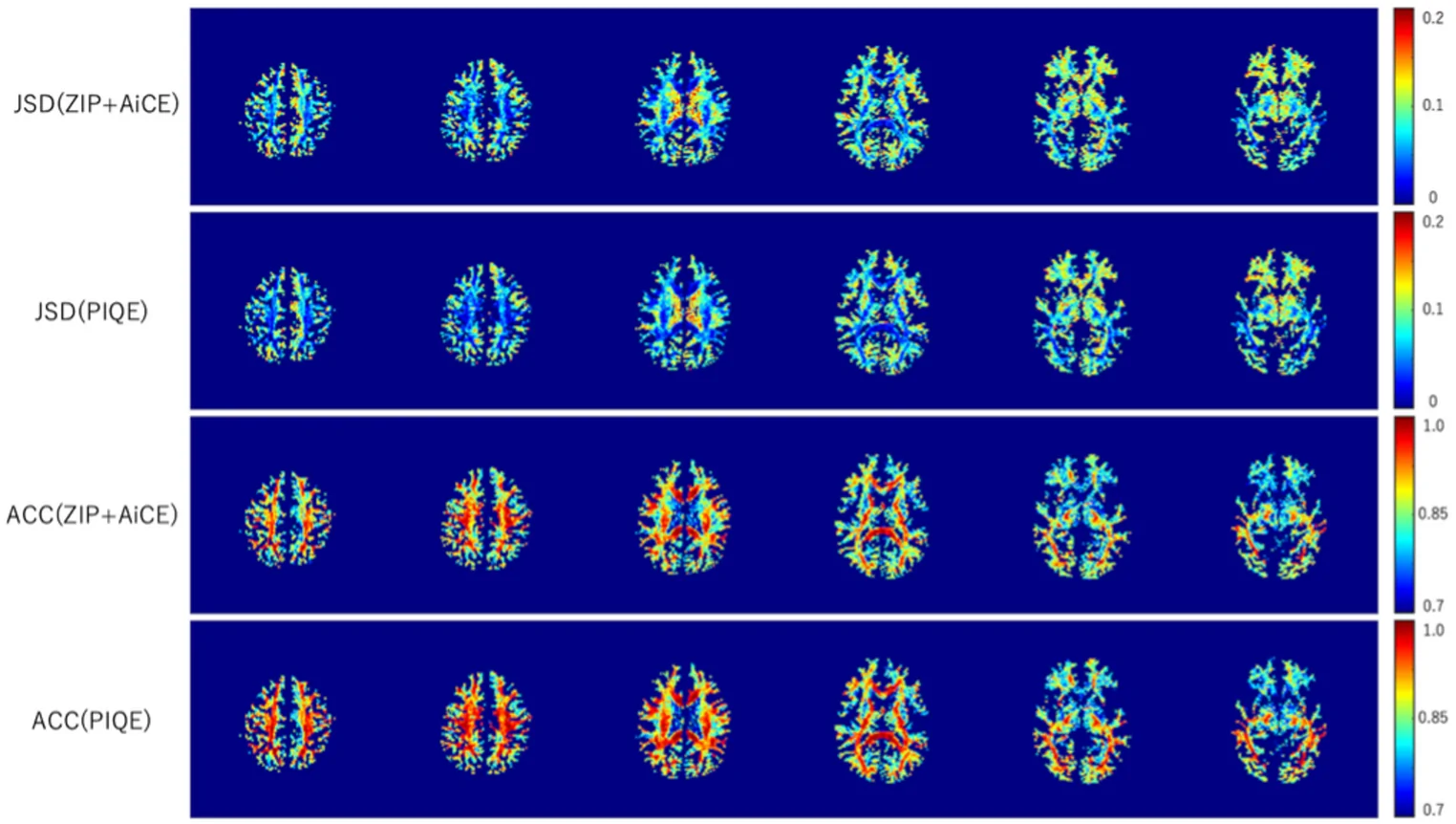
JSD and ACC maps for a representative subject

**Fig. 4:**
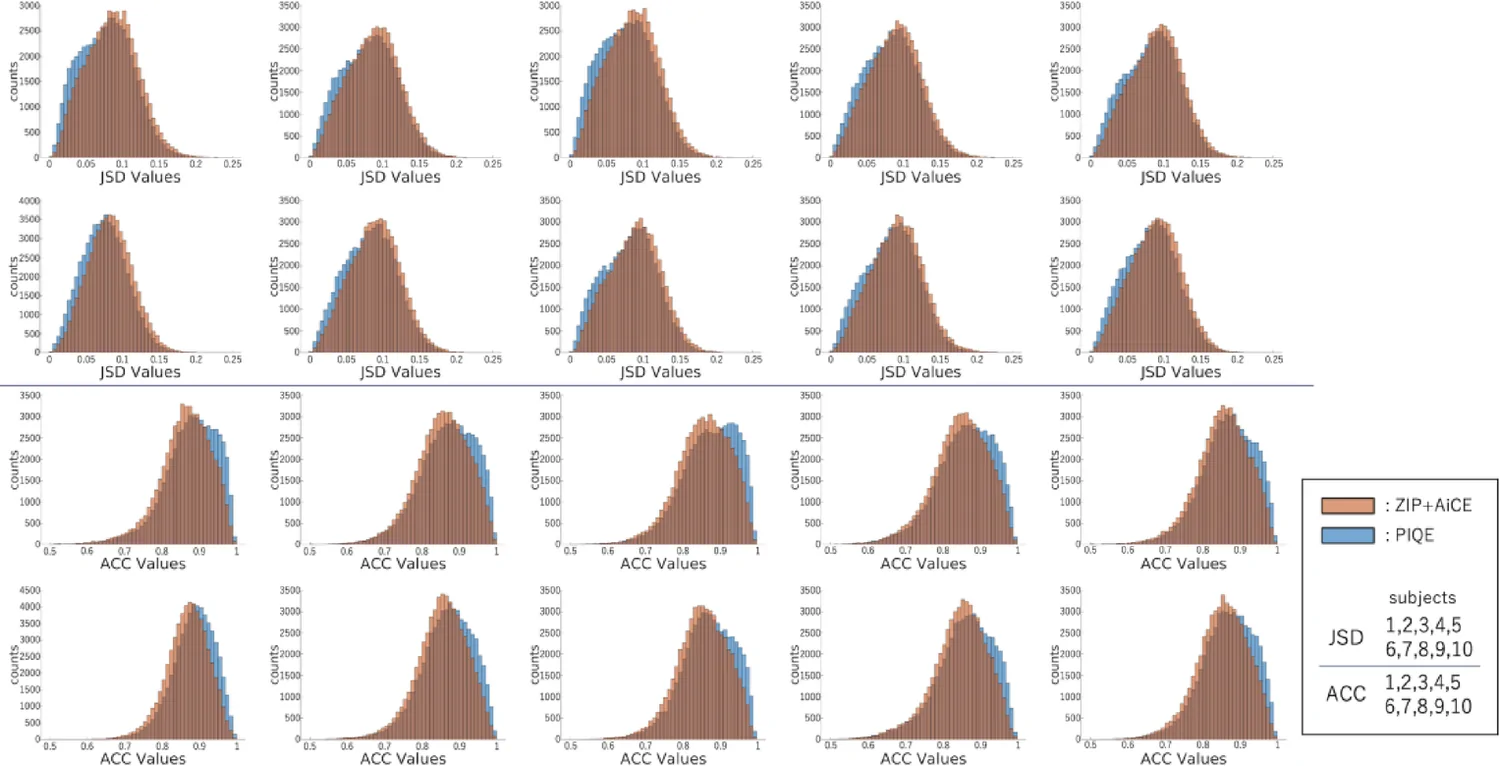
The distributions of JSD and ACC values obtained from each dataset

The b_0_ images, the isotropic DWIs, and the DWIs acquired in the first of the 64 MPG directions, reconstructed from the reference data, ZIP+AiCE data, and PIQE data, are shown for a representative subject (Fig. 2). The corresponding JSD and ACC maps within the white matter region for a representative subject are shown (Fig. 3). Furthermore, the distributions of JSD and ACC values obtained from each dataset are shown (Fig. 4). The mean values of JSD and ACC calculated within the white matter region for each subject are also presented (Table 1). In Fig. 4, although the distributions of ZIP+AiCE and PIQE largely overlap, PIQE consistently shows a systematic shift toward lower JSD and higher ACC values across all subjects. This tendency is also supported by the subject-wise mean value (Table 1), in which PIQE demonstrated lower JSD and higher ACC in all cases. The mean differences between the methods were approximately 0.007 for JSD and 0.014 for ACC. The Wilcoxon signed-rank test further confirmed that the differences in both JSD and ACC between the two methods were statistically significant (*p* < 0.05). The effect size, calculated as the rank-biserial correlation, was − 1.0 for JSD and + 1.0 for ACC (lower JSD and higher ACC for PIQE). This indicates that all subjects showed differences in the same direction, reflecting complete directional consistency, and offers a quantitative characterization of the observed differences.

Although the distributions largely overlap, PIQE shows a consistent shift toward lower JSD and higher ACC values compared to ZIP+AiCE.

**Table 1:**
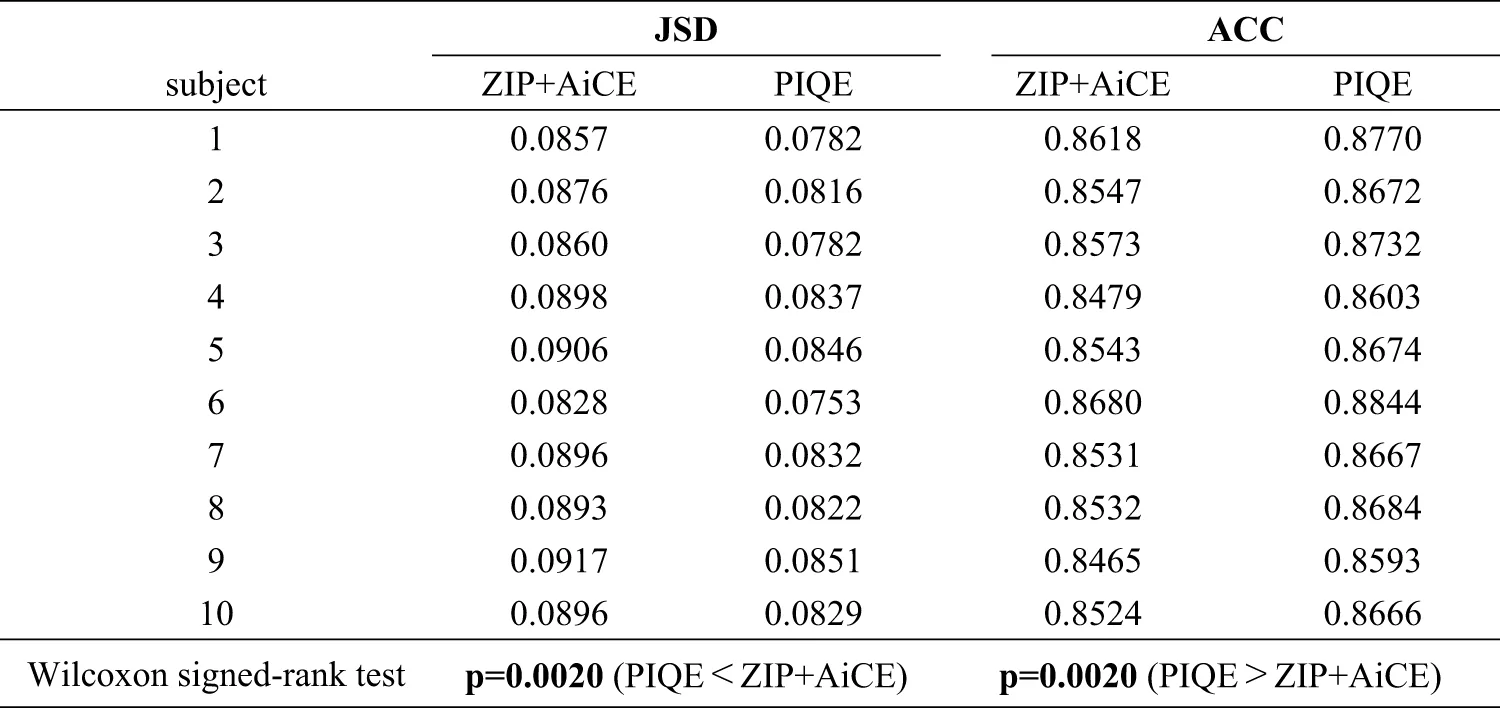
The mean JSD and ACC values calculated within the white matter region of each subject

The bold values indicate statistically significant differences (*p* < 0.05) between ZIP+AiCE and PIQE, highlighting the superior performance of PIQE compared to ZIP+AiCE

## Discussion

### Interpretation of quantitative results on reconstruction similarity to the reference data

In the present study, we evaluated the reconstruction similarity to the reference data of FOD in diffusion MRI by comparing a novel deep learning–based super-resolution method, PIQE, with the conventional approach, ZIP+AiCE. For the comparison, two quantitative metrics were employed: JSD, to assess the similarity of diffusion signal distributions, and ACC, to evaluate directional agreement. As a result, PIQE demonstrated statistically significant differences over ZIP+AiCE in both metrics, consistently demonstrated higher similarity to the reference data compared to ZIP+AiCE. These findings suggest that PIQE contributes not only to preserving the overall shape of the FOD distribution, but also, more notably, to enhancing directional similarity to the reference data. In the study by Tian et al. [18], a deep learning–based method called Super-Resolution for Diffusion Tensor Imaging (SRDTI) was proposed to generate super-resolved images from low-resolution DTI. In that previous study, diffusion tensor data obtained from the Human Connectome Project (HCP) were down sampled and used as input. The images reconstructed by SRDTI were shown to more closely resemble the original high-resolution data compared to those obtained using non–deep learning methods such as trilinear or spline interpolation. Although the methods and conventional comparisons differ from those in the present study, this report provides an example where AI-based super-resolution methods have demonstrated the potential to perform better than conventional reconstruction techniques. Although the statistical analysis demonstrated consistent improvement across all subjects, the absolute differences in JSD (approximately 0.007) and ACC (approximately 0.014) were relatively small. The clinical or practical significance of these differences remains uncertain, particularly in the context of downstream applications such as tractography and fixel-based analysis. Further studies are required to determine whether these quantitative improvements translate into meaningful differences in higher-order diffusion MRI analyses.

### Processing framework of PIQE and factors contributing to closer agreement with the reference

The results obtained in this study are considered to stem from the characteristics of the processing framework adopted by PIQE. PIQE employs a two-stage neural network, achieving image reconstruction with higher similarity to the reference data. This design philosophy of “separating processing objectives into stages” is structurally similar to the Multi-Stage Integration Network (MINet) proposed by Feng et al. [19]. MINet consists of the two stages to achieve super-resolution for multi-contrast MRI: While PIQE and MINet differ in their specific implementations and processing steps, they share the advantage of “controlling complex features and degradations that cannot be fully captured by a single processing step through a staged approach.

### The similarity of PIQE to the reference in FOD estimation

The physical limitations of ZIP are rooted in the zero-padding of high-frequency components in k-space, which is known to cause Gibbs phenomena (ringing artifacts) and subsequent smoothing in the spatial domain [20]. It is inferred that these physical effects cause ZIP+AiCE to smooth the peak structures of FODs, leading to unstable estimation of directional vectors. This observation is supported by the significantly higher similarity of PIQE with the reference in both JSD and ACC.

Although the 64 × 64 input data has an inherent SNR advantage over the 128 × 128 reference data, it suffers from substantial partial volume effects that reduce the similarity of FOD estimation to the reference data. In this study, the ‘super-resolution’ task performed by PIQE was evaluated strictly in terms of its ability to approximate the standard-resolution reference under identical scan time conditions. By leveraging a deep learning-based framework, PIQE yielded FOD profiles that showed statistically closer numeric agreement with the reference acquisition than ZIP+AiCE. This suggests that PIQE may reduce the structural smoothing inherent to low-resolution matrix acquisitions, leading to a higher similarity to the reference data under the fixed-scan-time protocol. However, whether this numeric closer agreement represents a genuine preservation of underlying biological microstructures remains to be verified.

Furthermore, in regions where FODs exhibit multiple peaks, even slight intensity differences or blurring can cause significant shifts in the principal directions [5]. In this study, “principal directions” refers to the dominant multiple orientations expressed within the FOD. As investigated by Tournier et al. [4], subtle differences in signal strength along the main direction directly affect the formation of FOD peaks. This revised analysis demonstrates that the observed numeric similarity to the reference data is consistently maintained even in the presence of complex directional variations and delicate structural changes.

### Adjustment of evaluation conditions and validity of comparison

To ensure a fair and rigorous comparison, we applied AiCE—an AI-based denoising technique—to the ZIP data to reduce differences in noise characteristics. Since both PIQE and ZIP+AiCE incorporate AI-based SNR improvement and rely on ZIP for initial processing, the performance gap primarily highlights the critical role of PIQE’s second neural network in providing structural correction. By assigning limited processing objectives to each stage, PIQE enhances learning efficiency and maintains high generalization performance, ultimately contributing to a closer alignment with the reference white matter profile under these experimental conditions.

### Limitations and future directions of the present study

A limitation of this study is that the clinical advantage of scan time reduction was not directly demonstrated, as the acquisition times for both protocols were identical. The primary focus of this work was strictly limited to a comparative technical evaluation of PIQE and ZIP+AiCE regarding their mathematical similarity to the acquired reference data. Although one might theoretically speculate that this low-resolution acquisition strategy could be beneficial in typical clinical settings where SNR or hardware capabilities are constrained, such clinical utility remains purely hypothetical. Since our experiment was conducted under a fixed-scan-time environment and did not evaluate actual scan-time reductions, conventional mid-range systems, or clinically relevant downstream tasks, any implications regarding the routine clinical advantage of PIQE must be interpreted with extreme caution. Further independent studies are strictly required to determine whether the observed technical similarities translate into meaningful benefits in clinical workflow or higher-order tract-specific metrics.

While effective for the HARDI data used here (b = 3000 s/mm^2^, 64 directions), the generalizability of PIQE to lower b-values or fewer directions (e.g., standard DTI) has not yet been verified. Additionally, PIQE was tested with fixed parameters (Denoise level = 3, zoom ratio = 2, DL Recon Adjust = 1.0, Edge Enhance = off); future work should evaluate how outcomes vary with different settings. For preprocessing, we uniformly applied standard MRtrix3-based preprocessing to all DWI datasets, including the Reference, ZIP, and PIQE data. In particular, such preprocessing was essential for the Reference data; thus, to ensure fair comparison under equivalent conditions, the same processing was also applied to the ZIP and PIQE data. Consequently, the ZIP+AiCE and PIQE data underwent double denoising—initially through their respective internal algorithms and subsequently via MRtrix3. In addition, the PIQE inherently includes the ringing artifact removal process. To address the potential effects of “double processing,” an additional analysis was conducted without applying the MRtrix3 denoise and ringing removal steps. The results showed that although the absolute JSD values were slightly higher and the ACC values slightly lower compared to the main analysis, the differences were consistently small across all subjects (approximately 0.003–0.007 for JSD and 0.01–0.015 for ACC) and did not alter the statistical significance (*p* = 0.0020) or the overall trend (Table 2), suggesting that the influence of double denoising on the conclusions of this study was limited.

**Table 2:**
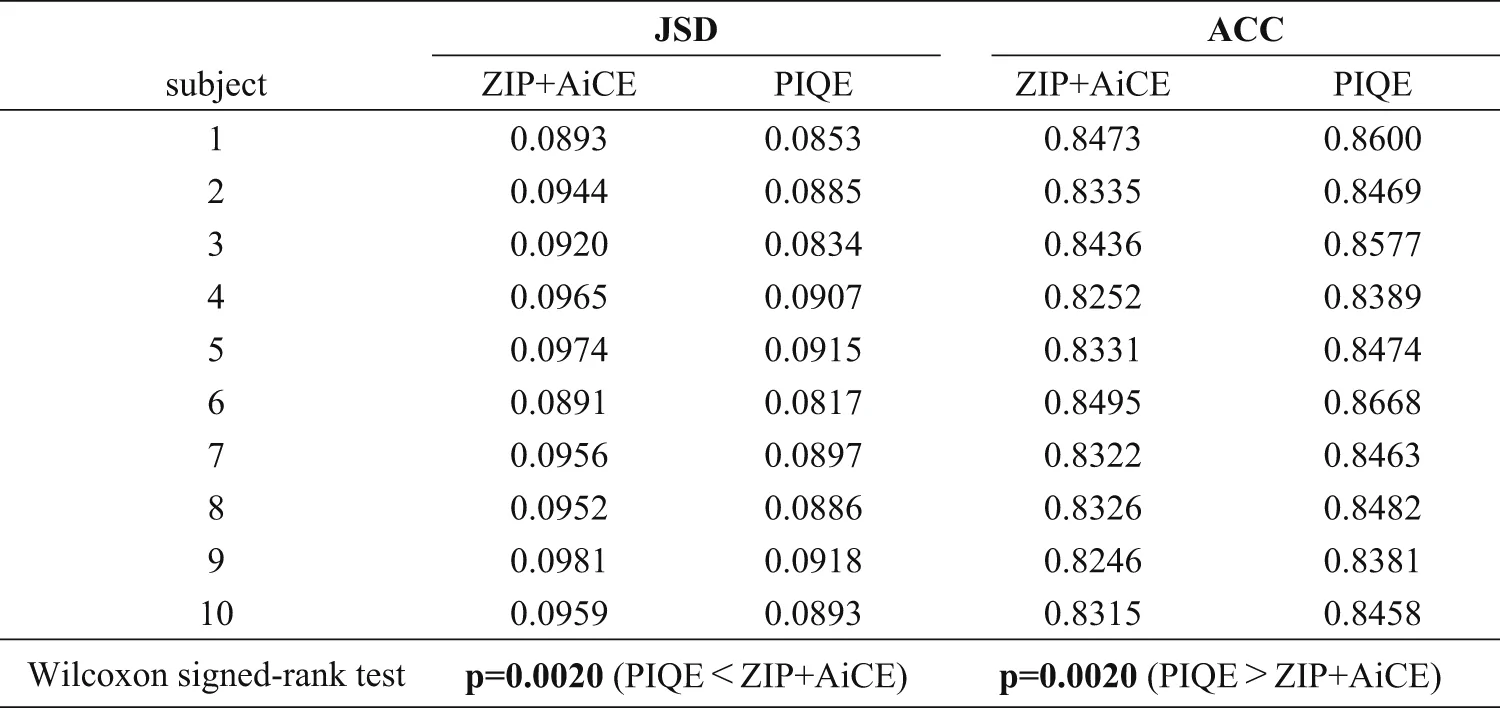
The mean JSD and ACC values calculated within the white matter region of each subject

The bold values indicate statistically significant differences (*p* < 0.05) between ZIP+AiCE and PIQE, highlighting the superior performance of PIQE compared to ZIP+AiCE

In this study, the response functions (RFs) were estimated individually for each reconstruction method to evaluate them under their optimal performance conditions, reflecting the distinct signal-to-noise and resolution profiles of ZIP+AiCE and PIQE. We acknowledge that this approach cannot completely rule out the possibility that slight variations in the estimated RF shapes may have confounded the FOD comparisons. However, additional analyses (Tables 1 and 2) demonstrated that the statistical significance of PIQE remained robust even when signal characteristics were intentionally altered. This consistency appears to suggest that the improvements in JSD and ACC are fundamentally driven by the inherent algorithmic differences between the methods rather than by minor fluctuations in the RFs. Consequently, while some RF variability may exist, it does not appear to be of a magnitude that would critically alter the primary conclusions regarding the higher similarity achieved by PIQE.

Beyond preprocessing-related factors, the SNR relationship between the low-resolution input data (64 × 64) and the standard-resolution reference data (128 × 128) differs from typical super-resolution scenarios where the target high-resolution images typically possess higher SNR. While this unique SNR profile could potentially influence similarity metrics, our results suggest that the restoration of spatial resolution is the primary factor in enhancing the consistency with the reference data.

It should also be noted that the “reference” dataset in this study was obtained through actual acquisition and cannot be regarded as a true ground truth. Due to the probabilistic nature of MR signal intensity and the presence of noise, the FOD estimated from the reference data inevitably contains uncertainty. Therefore, the present results represent the similarity or agreement with the reference data rather than the absolute accuracy of FOD estimation. This distinction should be taken into account when interpreting the findings. In addition, the use of a single-vendor system (Canon) for both the reconstruction method (PIQE) and the reference data may introduce system-specific biases, such as noise characteristics or point spread function. Consequently, the observed similarity between PIQE and the reference data may partially reflect shared system-specific properties, rather than solely reflecting differences in the recovery of underlying fiber structures. This limitation should be considered when interpreting the present findings. In addition, different FOD representation or normalization strategies may influence quantitative similarity metrics such as JSD. Furthermore, it is essential to note that the higher similarity achieved by PIQE demonstrated in this study is limited to the degree of numerical agreement in FOD reconstruction to the reference data, as assessed by JSD and ACC. A high degree of FOD similarity does not directly guarantee the reproducibility of results in higher-order analyses such as tractography or fixel-based analysis, nor does it ensure absolute consistency with parameters from other diffusion models or with the DWI signal intensity itself.

Another limitation of this study is that PIQE is a proprietary reconstruction method, and its internal implementation details, including training data, network architecture and loss functions, are not publicly disclosed. Consequently, the results obtained using PIQE cannot be independently reproduced or fully validated by external researchers, which may limit the transparency and generalizability of the findings. This issue should be considered when interpreting the results.

Furthermore, the number of subjects included in the present study was limited, and all participants were male, which may limit the generalizability of the findings. To generalize the statistical validity of the performance of PIQE, further studies involving more diverse populations and clinical datasets are warranted. Comparative analysis with other AI-based super-resolution methods (e.g., EDSR [21], SRCNN [22], GAN-based models [23]) would also help clarify the relative positioning and characteristics of PIQE.

## Conclusion

The present study showed that by applying the AI-based super-resolution technique PIQE to HARDI, the similarity of FOD estimation to the reference data of FOD in nerve fiber structure estimation is significantly increased compared to the conventional method ZIP+AiCE. Specifically, low-resolution diffusion data were reconstructed to standard resolution and compared with standard-resolution acquisitions. PIQE produced FOD estimates with higher directional agreement to the reference data than ZIP+AiCE. This characteristic resulted in higher similarity to the reference data; however, its impact on downstream diffusion MRI analyses was not evaluated in this study and remains to be determined.

## Data Availability

The data supporting the findings of this study are available from the corresponding author upon reasonable request.
